# Spatiotemporal analysis of dengue fever in Burkina Faso from 2016 to 2019

**DOI:** 10.1186/s12889-022-12820-x

**Published:** 2022-03-08

**Authors:** Cheick Ahmed Ouattara, Seydou Traore, Ibrahim Sangare, Tiandiogo Isidore Traore, Ziemlé Clément Meda, Léon G. Blaise Savadogo

**Affiliations:** 1 NAZI BONI University, Centre Hospitalier Universitaire Souro Sanou, Bobo-Dioulasso, Burkina Faso; 2 Centre Hospitalier Universitaire Souro Sanou, Bobo-Dioulasso, Burkina Faso; 3 NAZI BONI University, Bobo-Dioulasso, Burkina Faso

**Keywords:** Dengue, Burkina Faso, Incidence, Excess risk, Cluster

## Abstract

**Background:**

Burkina Faso experienced an epidemic resurgence of dengue in 2016, which led to the implementation of several control strategies. In order to allow a better adaptation of these strategies, we studied the spatio-temporal distribution of dengue.

**Methods:**

Monthly dengue cases from 2016 to 2019, aggregated at the health district level, were used to map the crude incidence, excess risk, and smoothed incidence of dengue in Burkina Faso with GeoDa software. A Kulldoff scan on Satscan software was then used to identify spatio-temporal clustering of cases.

**Results:**

The results show that the distribution of dengue fever across the health districts of Burkina Faso is heterogeneous. Dengue was considered non-endemic in 9 out of the 70 health districts, minimally endemic in 45 districts (< 10 incidences), moderately endemic (10-100 incidences) in 12 districts, and highly endemic (> 100 incidences) in 4 districts. The main cluster covered the health districts of Baskuy, Nongr-massom, Sig-noghin, Boulmiougou, and Bogodogo. The months of October and November corresponded to the peak of cases and a significant temporal cluster in 2017.

**Conclusion:**

This study identified the spatial and temporal clustering of dengue cases in Burkina Faso. These results may help to develop better preventive strategies.

**Supplementary Information:**

The online version contains supplementary material available at 10.1186/s12889-022-12820-x.

## Background

Dengue is an arbovirus infection caused by a Flaviviridae family’s virus, of which there are four serotypes (Dengue Virus 1, 2, 3 and 4). It is transmitted to humans by the bite of infected female mosquitoes of the genus Aedes (*Aedes aegypti* and *Aedes albopictus*) [1]. The World Health Organization distinguishes symptomatic forms of dengue into dengue with or without warning signs, a mild and common form, and severe dengue with bleeding complications, signs of shock or visceral failure [2]. Classified as a neglected tropical disease, dengue has been experiencing epidemic resurgence in recent years and is present in more than 128 countries [1, 3]. Its social and economic impact is increasingly important.

The first case of dengue (probably base on dengue-like syndrome) in Burkina Faso was reported in 1925. The country experienced epidemics in 1982 with patients positive for specific anti-Dengue IgM antibodies and virus isolation in cell culture, 2013, 2016 and then 2017 [4–6].

Dengue was then included in the country’s list of priority diseases in 2016, and integrated into the list of diseases under epidemiological surveillance. Various intervention programs ranging from diagnostic capacity building to community-based interventions have been implemented to control dengue [7–9].

Previous studies have shown that the spatial distribution of dengue can be quite heterogeneous within a country and even at subnational scales. Seasonality of dengue transmission mainly influenced by climatic factors has also been reported [10–13].

Knowledge of the spatial and temporal epidemiology of dengue over time in Burkina Faso are essential for adapting dengue control programs. To our knowledge, there are no studies that have explored this. The objective of the present study is to assess and map the spatio-temporal trends of dengue cases in Burkina Faso at the health district level.

## Methods

### Study area [14]

**Fig. 1:**
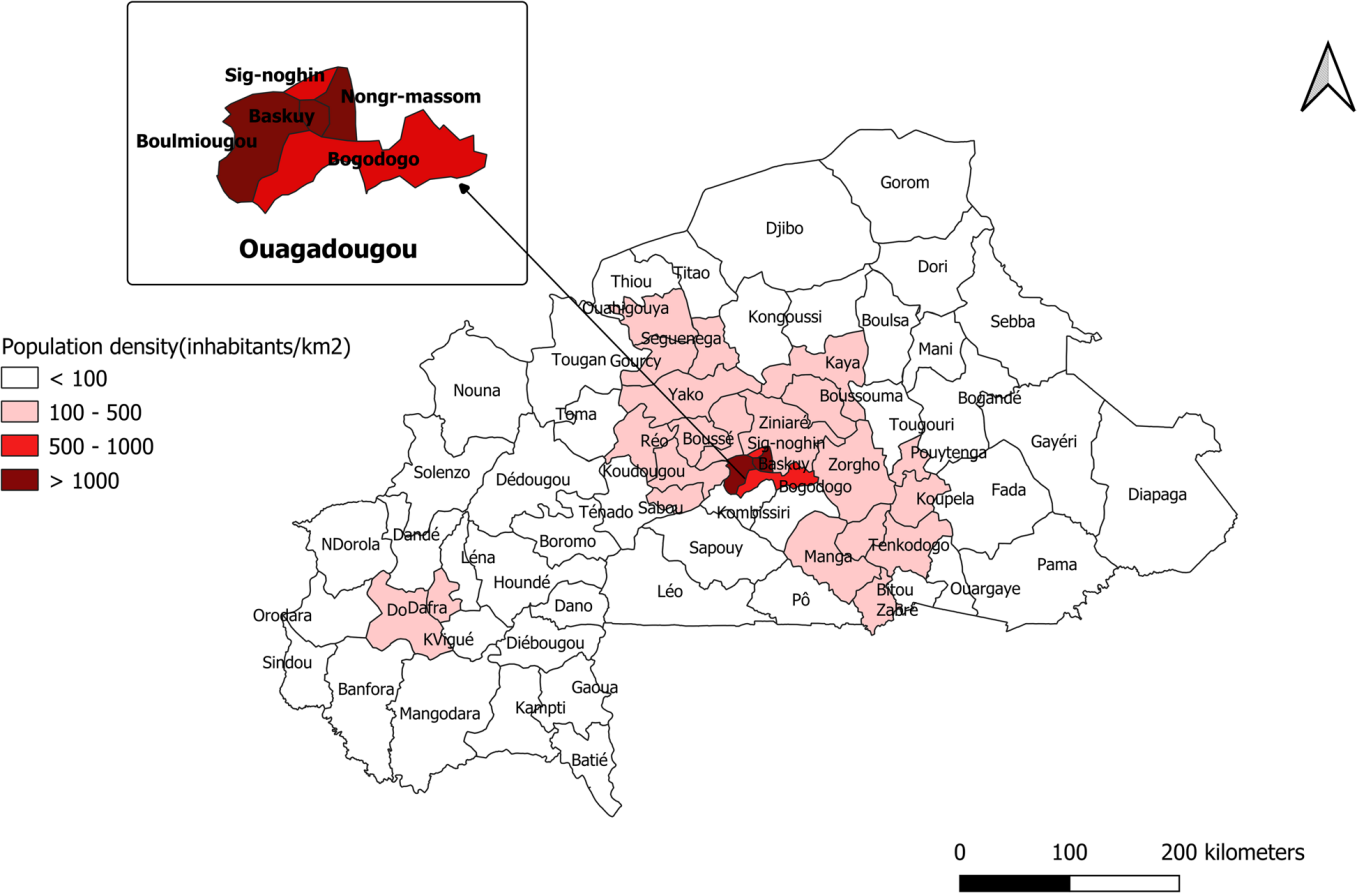
Study area: Health districts in Burkina Faso with population density indicated

Burkina Faso is located between 8° to 16° north in latitude and in longitudes 6° west and 3° east. It is administratively divided into thirteen regions and forty-five provinces. The health system has a pyramidal organization with a central, intermediate and peripheral level. The peripheral level is made up of seventy health districts. A province may have several health districts, as shown in Fig. 1. The analyses were conducted at the health district level.

### Data source

The dengue cases used in this study were extracted from the health statistics yearbooks of Burkina Faso. All health data from 1 year are compiled and validated by the Ministry of Health of Burkina Faso and published annually in the last quarter of the following year. Dengue cases data are registered and reported in health statistics yearbooks of Burkina Faso since 2016. Dengue cases were diagnosed based on a rapid diagnostic test (Immunoglobulin M and/or Immunoglobulin G and/or NS1 dengue antigen positive), confirmed or not by Polymerase Chain Reaction analysis in public and private health facilities. The statistical health yearbooks also provide the populations of each health district based on population projections made by the National Institute of Statistics and Demography. The shapefile at the health district level was produced by the Burkina Geographic Institute and is available online [15]. All data use in this study is available in supplementary data (see Additional file 1).

### GIS mapping and smoothing [10]

We used choropleth mapping as a technique to analyze the incidence of dengue in Burkina Faso. We first calculated the annual incidence per 100,000 inhabitants for each health district by summing all cases notified per year in the health facilities of each district and dividing by the population of the corresponding district. Then, we calculated the average annual incidence by dividing the sum of the annual incidences from 2016 to 2019 for each health district by four.

This resulted in a four-year average annual dengue incidence map, an excess risk map, and a spatially smoothed distribution map using an empirical Bayes approach. The health districts have areas and populations that vary greatly in size. This poses the problem of small numbers in the risk mapping approach. This small number effect makes risk estimation unstable. That is, one case more or less can cause a large variation in the estimated risk. The effect of this is that the extreme values on a classic chloropleth map of estimated risks will display a tendency to be concentrated in the smaller areas. Smoothing here is suitable because it allows to get rid of the “small numbers” problem, to discover unexpected gradients and to reduce unusual or outlying values [16]. Mapping and smoothing were done with GeoDa 1.18.0 software.

### Spatiotemporal cluster analysis [10]

Dengue clusters were identified with SaTScan version 9.6 software [17, 18] by purely spatial and purely temporal analysis. This approach seeks to group different neighboring statistical units into potential clusters using a geographically shifting window. By comparing observed cases to expected cases inside and outside a window, a cluster is identified if the observed cases exceed the expected cases [19].

## Results

### Spatial and temporal distribution of dengue in Burkina Faso

**Fig. 2:**
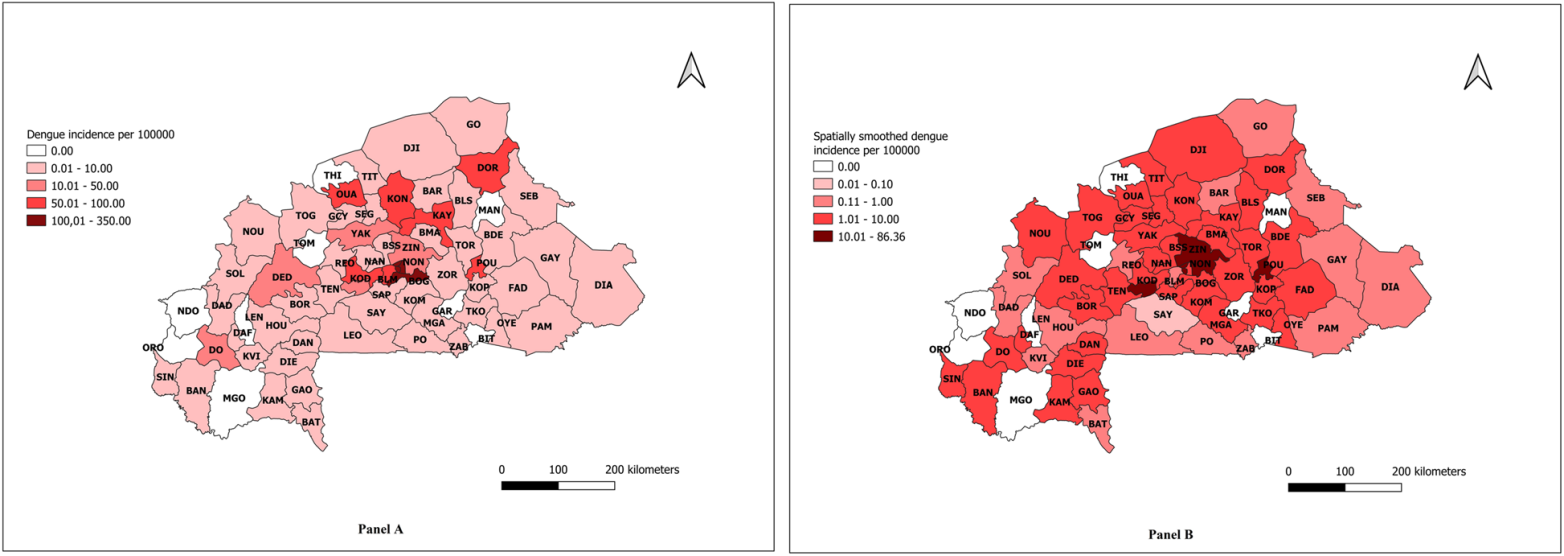
Annual average incidence (panel **A**) and spatially smoothed incidence (panel **B**) of dengue fever in Burkina Faso (2016-2019)

From 2016 to 2019, 24,526 cases of dengue were reported in Burkina Faso. The mean annual incidence ranged per health district from 0 to 350 per 100,000 inhabitants (Fig. 2A). Of 70 health districts, 9 were considered nonendemic with zero annual incidence. Forty-five districts were low endemic (< 10 incidences), 12 were moderately endemic (10-100 incidences), and 4 were highly endemic (> 100 incidences).

The Sig-Noghin district had the highest level of endemicity with a total of 2833 cases for an average annual incidence of 350 per 100,000 inhabitants.

**Fig. 3:**
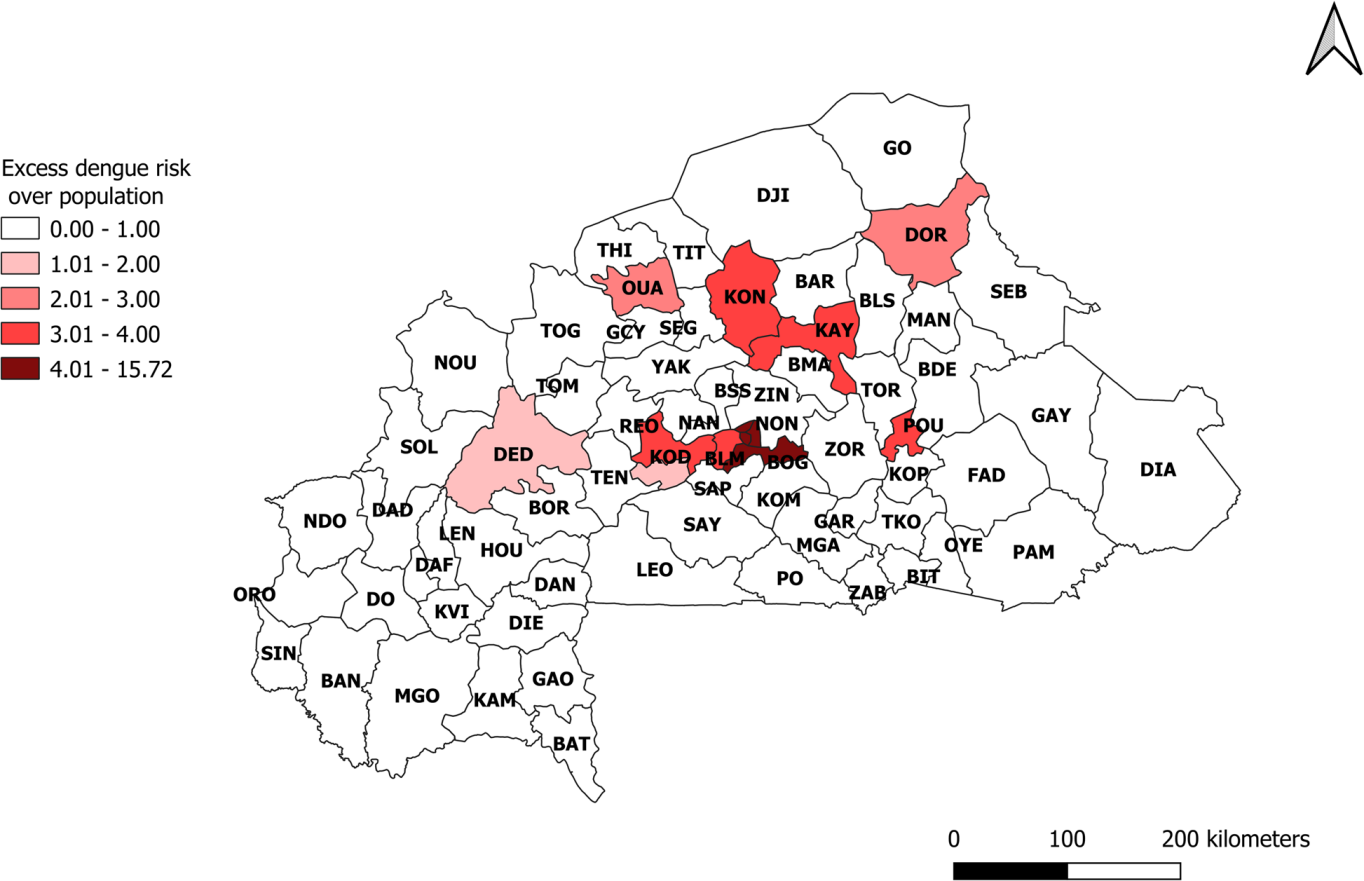
Excess hazard map of dengue fever in Burkina Faso (2016-2019)

Figure 3 presents the distribution of dengue excess risk in Burkina Faso (2016-2019). It shows that 22 health districts had an excess risk, while the risk was lower than expected in 48 health districts. The intensity of the risk is proportional to the accentuation of the red color. Five districts had a significant excess risk. In order of importance, these were the health districts of Pouytenga, Sabou, Sig-noghin, Ziniaré and Baskuy. The health district of Boulsa had the lowest excess risk.

Figure 2B shows a better distribution of dengue incidence at the health district level because it is smoothed and therefore without spatial autocorrelation. It shows clearly that the contiguous zone of the health districts of Sig-noghin, Baskuy and Ziniaré is an area of high dengue incidence. The health districts of Pouytenga and Sabou appear to have an equally high incidence, in contrast to the difference in incidence shown in Fig. 2A.

**Fig. 4:**
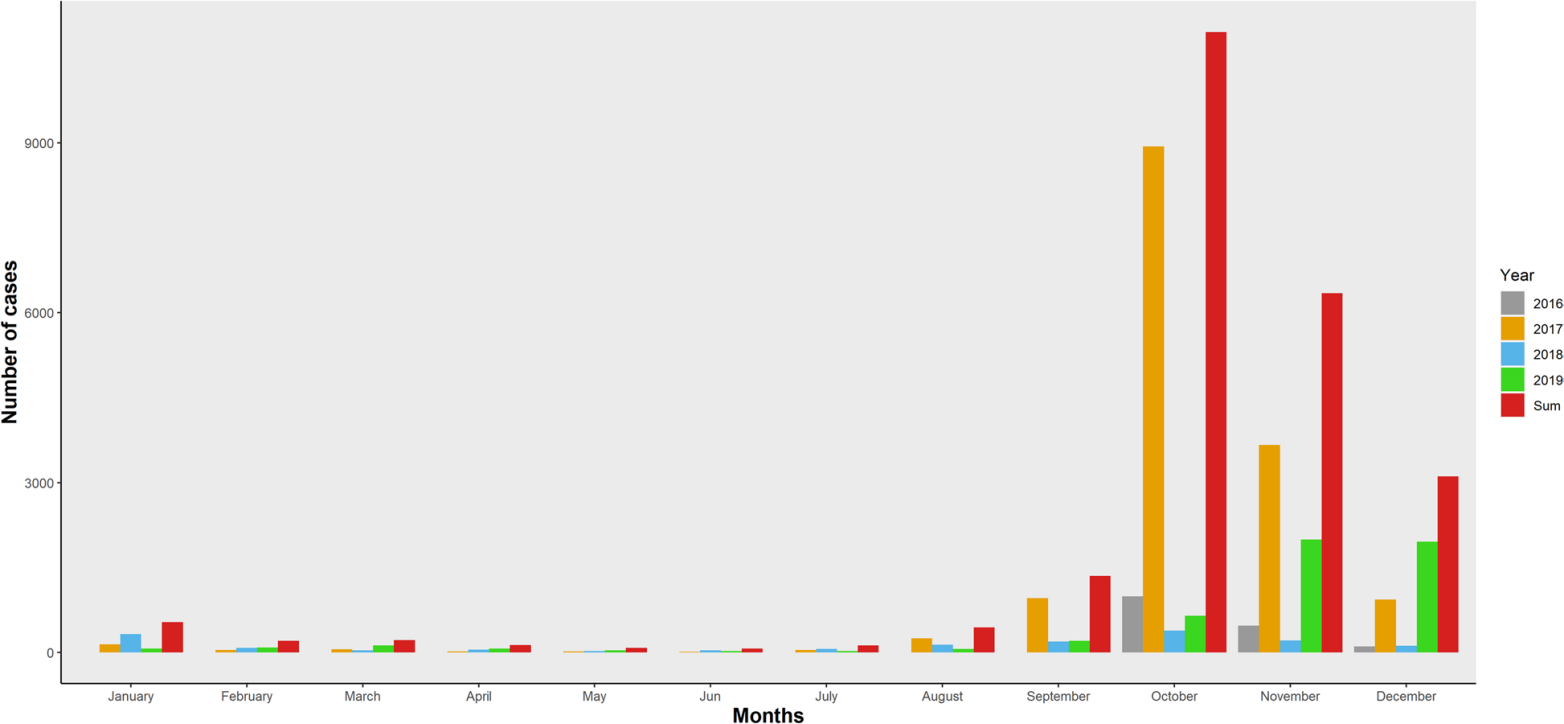
Monthly distribution of dengue fever in Burkina Faso (2016-2019)

Figure 4 shows that October was the peak month for dengue cases throughout 2016-2019 including during the 2016 and 2017 outbreaks.

### Distribution of dengue clusters

#### Spatial clusters

**Fig. 5:**
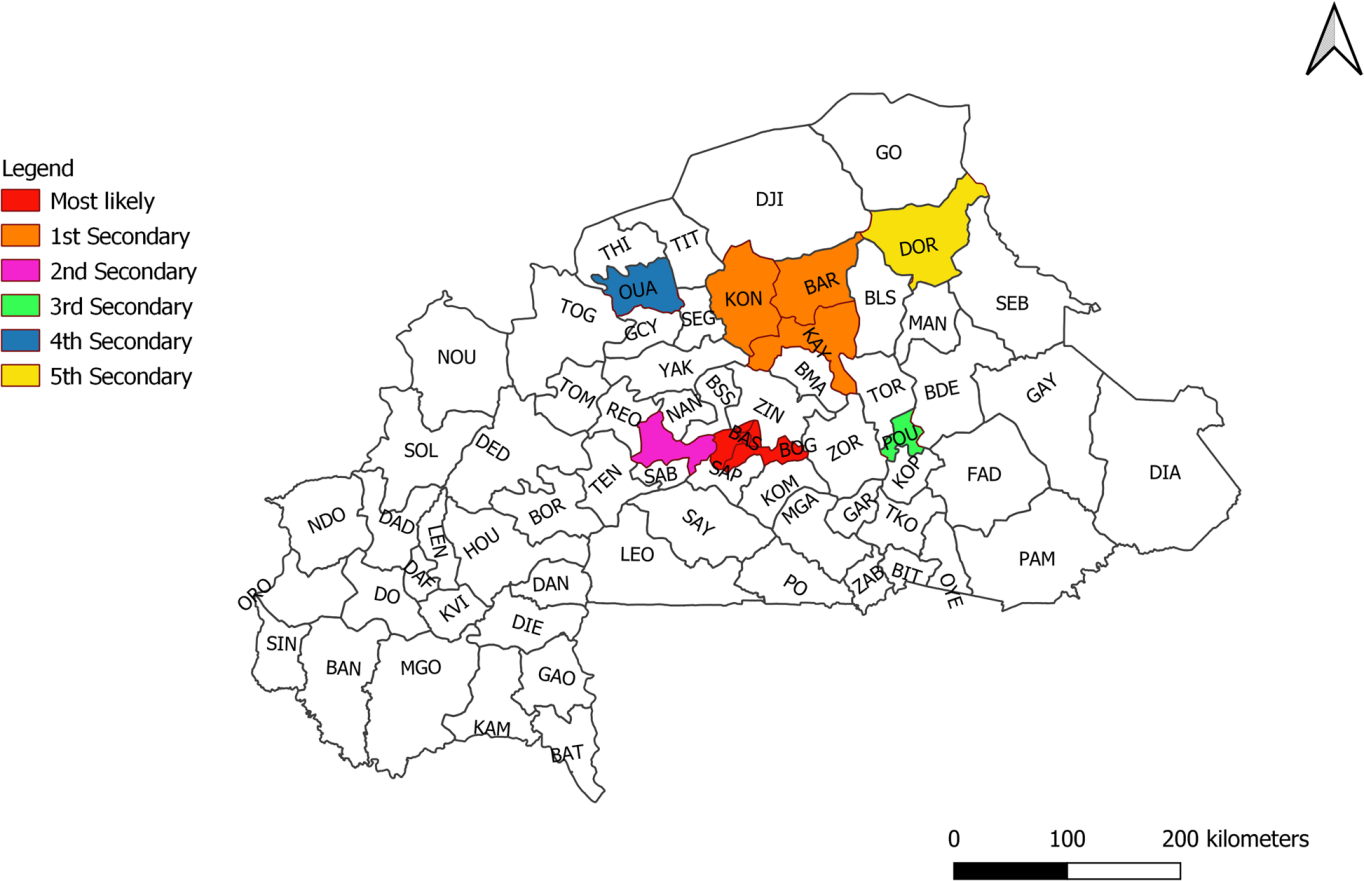
Distribution of spatial clusters of dengue fever in Burkina Faso (2016-2019)

The purely spatial analysis of dengue cases from 2016 to 2019 identified one main cluster and five secondary clusters (Fig. 5). The main cluster covers the health districts of Baskuy, Nongr-massom, Sig-noghin, Boulmiougou, and Bogodogo. They correspond to the boundaries of the central region.

The health districts of Barsalogo, Kaya, and Kongoussi in the North- Central region represented the first secondary cluster. The other secondary clusters were the health districts of Koudougou, Pouytenga, Ouahigouya and Dori. Table 1 provides the detailed result of the spatial analysis.

**Table 1:** Dengue cluster (2016 – 2019) based on purely spatial analysis under the Poisson Discrete probability model

District	Cluster type	LLR	P	Observed cases	Expected cases	Relative risk
Baskuy, Nongr-massom, Sig-noghin, Boulmiougou, Bogodogo	Most likely	16,901.66	< 0.001	16,091	3485.93	11.51
Barsalogo, Kaya, Kongoussi	1st Secondary	595.99	< 0.001	2541	1207.42	2.23
Koudougou	2nd Secondary	304.65	< 0.001	1099	476.05	2.37
Pouytenga	3rd Secondary	284.11	< 0.001	729	261.68	2.84
Ouahigouya	4th Secondary	140.825483	< 0.001	811	425.36	1.94
Dori	5th Secondary	138.218281	< 0.001	846	453.50	1.87

#### Temporal clusters

**Fig. 6:**
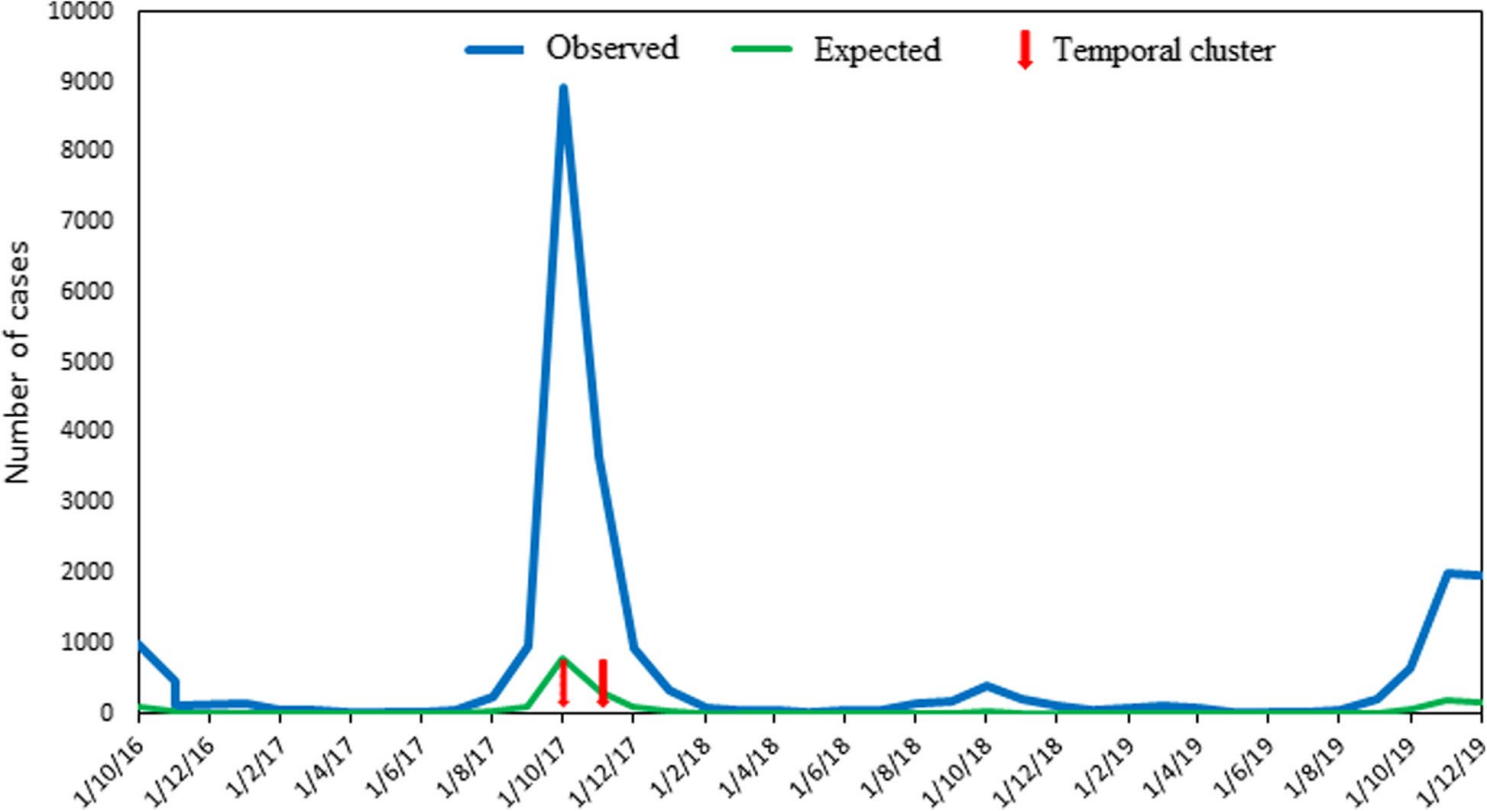
Temporal cluster of dengue fever in Burkina Faso (2016-2019)

The purely temporal analysis identified October and November in 2017 (Fig. 6) as a highly significant temporal group (Observed = 12,605, Expected = 1132.48, Relative Risk = 22.80, Log-Likelihood Ratio = 22,527.13, *p* < 0.001). Observed dengue cases were also high at the same period in 2016, 2018, and 2019, but were not identified as clusters.

## Discussion

In this study, we mapped the crude incidence, excess risk, and smoothed incidence of dengue at the health district level in Burkina Faso. We also identified significant spatial and temporal groupings of dengue cases.

To our knowledge, this is the first application of such a methodology to dengue data in Burkina Faso. This methodological approach is a strength of this study, as it is adapted for the analysis of aggregated data of a disease with low incidence or when the analysis involves spatial aggregation units of very heterogeneous size, small in places [10, 16]. Smoothing highlights unexpected gradients and reduces outliers [20] presenting a better incidence distribution mapping.

We used passive surveillance data for dengue in this study. The main limitations of this study are related to this. Indeed, this type of data is subject to under-reporting of cases. Also, aggregation at the district level, which corresponds to large geographical units, does not allow capturing heterogeneity of distribution at lower scales.

The results showed that the central region is the most vulnerable in the country. It covers the capital city, Ouagadougou, which is the most populated and urbanized city in the country. Dengue seems to be more urban because apart from the health district of Barsalgho, all dengue clusters are urban districts.

On one hand urbanization favors the development of artificial containers, development sites for *Aedes aegypti* dengue vectors. The high density of the population and their great mobility favor in turn the explosion of dengue cases. The high inter-mobility and contiguity with Ouagadougou could explain the excess of cases found in the health district of Ziniaré, which is located 35 km away and where different activities bring together actors from Ouagadougou. The same is true for the health districts of Sabou and Dédougou in relation with Koudougou.

On the other hand, dengue cases in non-urban areas are likely to be underestimated due not only to poor diagnostic accessibility but also to missed diagnoses. Indeed, many cases of fever are considered as malaria and treated as such in these areas without biologic confirmation.

The peak of cases has always been observed between October and November with a relative stability outside this period. This period follows the rainy season (May - October) in Burkina Faso. This seasonality in dengue transmission has been reported by several authors. With a time lag, there is a significant link between dengue incidence and climatic factors such as temperature and rainfall. In the life cycle of *Aedes Aegypti*, the main dengue vector in Africa, studies have shown that at optimal average temperatures (< 18 °C) an increase in temperature increases the incidence of dengue by shortening the development period of *Aedes aegypti* larvae and improving their blood feeding and oviposition, whereas at high average temperatures (> 18 °C) an increase in temperature will reduce the survival of *Aedes* and thus the transmission of dengue [21–23].

Large amounts of rainfall may result in the short-term removal of *Aedes aegypti* eggs and larvae from potential containers, but residual water may create longer-term breeding habitats [24–26].

The results of this study suggest strengthening dengue control interventions in urban settings and in the last quarter of the year. A study in the city of Ouagadougou at the level of health facility coverage areas and administrative sectors, exploring other risk factors for dengue, will complement the present study. Active surveillance in rural areas may provide a more reliable estimate of the burden of dengue in these areas.

## Conclusion

This study identified health districts with a high risk of dengue transmission in Burkina Faso. They are concentrated around the central region or share a relatively high mobility with it. The temporal dynamics of cases are seasonal with peaks between October and November. These results could allow the implementation of an intervention program targeting high risk districts and or the post winter period. They also open research perspectives focused on the environmental and socio-behavioral determinants of these spatio-temporal clusters.

## Supplementary Information

**Additional file 1.** Dengue annual cases by health district in Burkina Faso. This database contains by year for each health district in Burkina Faso, the dengue cases, the population, the average and smoothed incidences and excess hazard risk of dengue.

## Data Availability

Annual dengue cases by health district analysed during this study are included in this published article [and its supplementary information files] and are available in the annual statistical yearbooks accessible online at http://cns.bf/spip.php?id_rubrique=17&page=publdetails.
